# The Relationship between DUGBE Virus Infection and Autophagy in Epithelial Cells

**DOI:** 10.3390/v14102230

**Published:** 2022-10-11

**Authors:** Marie Moroso, Aurore Rozières, Pauline Verlhac, Florence Komurian-Pradel, Olivier Ferraris, Christophe N. Peyrefitte, Glaucia Paranhos-Baccalà, Christophe Viret, Mathias Faure

**Affiliations:** 1 CIRI, Centre International de Recherche en Infectiologie, Univ Lyon, Inserm U1111, Université Claude Bernard Lyon 1, CNRS, UMR5308, ENS de Lyon, F-69007 Lyon, France; 2 Emerging Pathogens Laboratory, Fondation Mérieux, F-69002 Lyon, France; 3 Biomedical Research Institute of the French Army (IRBA), F-91220 Bretigny-sur-Orge, France; 4 Equipe Labellisée par la Fondation pour la Recherche Médicale, 54 Rue de Varenne, F-75007 Paris, France

**Keywords:** Dugbe orthonairovirus, Crimean–Congo hemorrhagic fever orthonairovirus, autophagy, MAP1LC3 lipidation, viral infection, epithelial cells

## Abstract

Dugbe orthonairovirus (DUGV) is a tick-borne arbovirus within the order *Bunyavirales.* Although displaying mild pathogenic potential, DUGV is genetically related to the Crimean–Congo hemorrhagic fever virus (CCHFV), another orthonairovirus that causes severe liver dysfunction and hemorrhagic fever with a high mortality rate in humans. As we previously observed that CCHFV infection could massively recruit and lipidate MAP1LC3 (LC3), a core factor involved in the autophagic degradation of cytosolic components, we asked whether DUGV infection also substantially impacts the autophagy machinery in epithelial cells. We observed that DUGV infection does impose LC3 lipidation in cultured hepatocytes. DUGV infection also caused an upregulation of the *MAP1LC3* and *SQSTM1/p62* transcript levels, which were, however, more moderate than those seen during CCHFV infection. In contrast, unlike during CCHFV infection, the modulation of core autophagy factors could influence both LC3 lipidation and viral particle production: the silencing of *ATG5* and/or *ATG7* diminished the induction of LC3 lipidation and slightly upregulated the level of infectious DUGV particle production. Overall, the results are compatible with the notion that in epithelial cells infected with DUGV in vitro, the autophagy machinery may be recruited to exert a certain level of restriction on viral replication. Thus, the relationship between DUGV infection and autophagy in epithelial cells appears to present both similarities and distinctions with that seen during CCHFV infection.

## 1. Introduction

Macroautophagy, commonly called autophagy, is a conserved multi-step process that constitutively participates in the maintenance of cell homeostasis by directing senescent and abnormal cytosolic components to lysosomal degradation. It involves the biogenesis of a membrane element called the phagophore that subsequently elongates and closes to form a double-membraned compartment named the autophagosome. Cytosolic cargoes that have become encapsulated into autophagosomes are degraded upon the fusion of autophagosomes with hydrolytic vesicles of the endo-lysosomal pathway. The overall process, termed the autophagy flux, relies on the engagement of multiple autophagy-related (ATG) proteins [1,2]. The targeting of cargo to be degraded by autophagy can be highly specific. In such cases, ubiquitinated or non-ubiquitinated cargoes are selectively bound by so-called autophagy receptors that also interact with lipidated members of the ATG8 family for anchoring on the phagophore membrane [3,4]. A prototypic example of a selective autophagy receptor is sequestosome 1 (SQSTM1)/p62, which can, for instance, mediate the autophagic targeting of protein aggregates and, interestingly, can itself be subjected to autophagic degradation as it is a long-lived protein [5,6]. Among the best studied ATG8 factors are the microtubule-associated protein 1 light chain 3 proteins (MAP1LC3 or LC3) [7], whose lipidated forms serve as molecular markers for both autophagosome identification and the overall autophagic activity of cells. Besides its instrumental role in cell homeostasis maintenance, autophagy is an integral part of cell autonomous defense mechanisms against microbes capable of invading the cytosol of host cells (xenophagy). This is best illustrated by the fact that multiple pathogens have evolved the means to escape, antagonize, or manipulate autophagy [8,9]. In the case of viruses, autophagy can contribute to resistance by selectively degrading viral particles/viral components or by positively regulating type-I interferon production [10,11].

Crimean–Congo hemorrhagic fever virus (CCHFV) is a negative-sense RNA virus of the Nairoviridae family [12]. CCHFV can be efficiently transmitted by common ticks of the Hyalomma genus that also act as a reservoir for the virus [13]. CCHFV, classified as an agent of biosafety level (BSL) 4, causes mild-to-severe viral hemorrhagic fevers that represent an important threat to public health due to: (I) an elevated mortality rate in humans (10–40%) [14,15]; (II) a variety of modalities of transmission (from tick-to-human and animal, animal-to-human, and human-to-human); and (III) an increasing number of geographical areas affected by CCHFV outbreaks. CCHF is indeed considered endemic in about 30 countries [16,17]. Currently, no efficient treatments or vaccines are available. Because little is known about how CCHFV causes hemorrhagic fever and due to the significant spread of the virus, CCHF has for several years been on the list of diseases and pathogens prioritized by the World Health Organization for research and development in public health emergency contexts.

DUGBE orthonairovirus (DUGV) is another tick-borne arbovirus whose genome is made of a tripartite, single-stranded RNA of negative polarity. DUGV has zoonotic potential and can cause clinical manifestations in humans, although its impact on human health is clearly moderate relative to that of CCHFV [18,19,20]. Besides ticks, DUGV appears capable of infecting various hosts including distinct arthropods, ruminants, rodents, monkeys, and birds [21,22]. DUGV and CCHFV have been classified in the Nairobi sheep disease orthonairovirus (NSDV) and CCHFV serogroups, respectively [23,24,25], with possible antibody cross-reactivities in some immunoassays [26,27]. However, our knowledge of human cell infection by DUGV remains very limited.

As DUGV is genetically and antigenically related to CCHFV [28,29], dissecting the features of DUGV infection in epithelial cells may be helpful to better understand CCHFV infection itself [30,31]. Because we previously characterized the relationship between CCHFV infection and the autophagic activity of cultured epithelial cells [32], we decided to examine to what extent DUGV shares some features with CCHFV in this regard.

## 2. Materials and Methods

### 2.1. Ethics Statement

The experiments reported in this article were performed at Biological Safety Level 2 in accordance with the regulations set forth by the national French committee of genetics (commission de génie génétique).

### 2.2. Cell Culture

Huh7, HepG2, and VeroE6 cells were maintained in DMEM (Life Technologies, Courtaboeuf, France, 31966-021) supplemented with 50 U/mL of penicillin–streptomycin (Life Technologies, 15070-063) and non-essential amino acids for Huh7 cells (Life Technologies, 11140-035). HeLa and GFP-LC3-HeLa cells [33] were maintained in RPMI 1640 (Life Technologies, 61870-010) with 50 µg/mL gentamicin. All media were supplemented with 10% fetal bovine serum (FBS; Life Technologies, 10270-106) and maintained at 37 °C and 5% CO_2_.

### 2.3. Virus Strains and Viral Particle Titration

Cells were infected using DUGV isolate IbH 11480 (obtained from Pasteur Institute) produced in VeroE6 cells. The absence of Mycoplasma from cells and virus aliquots was confirmed using the MycoAlert Mycoplasma Detection kit (Lonza, Basel, Switzerland, LT07-710). Infections were carried out at two multiplicities of infection (MOI) for 1 h in DMEM media without FBS before adding complete media. After the indicated period of time of infection, infectious viral particles were quantified by limiting dilution in confluent Vero cells; cells were infected with serial dilutions of supernatants of virus-infected cells for 1 h. DMEM supplemented with 2.5% final FBS and 3.2% carboxymethylcellulose (VWR, Rosny-sous-Bois, France, 22525.296) was then added and cells were maintained at 37 °C, 5% CO_2_ for 5 days. VeroE6 cells were then fixed using 4% paraformaldehyde (Sigma, St Quentin Falavier, France, 252549) and incubated with a mouse hyper-immunized ascetic fluid specific to DUGV (obtained from the institut de recherche biomédicale de l’armée (IRBA)) for 1 h, followed by a 45 min incubation with a goat anti-mouse peroxidase (HRP)-conjugated antibody (Jackson ImmunoResearch, Cambridge, UK, 115-035-146) at 37 °C, 5% CO_2_. Foci of infected cells were detected using 3.3′-diaminobenzidine (Sigma, D4293). Titrations were determined as FFU/mL.

### 2.4. siRNA Transfection

Cells (0.1 × 10^6^) were plated in 6-well plates 24 h prior to transfection with 100 pmol siRNA/well (Stealth siRNA for Life Technologies) using Lipofectamine RNAiMAX (Life Technologies, 13800-075) according to the manufacturer’s instructions. Media were changed 4 h later using DMEM containing 2.5% FBS and cells were virally infected 48 h after siRNA transfection. Protein expression level was assessed by Western blot 2 days post-transfection.

### 2.5. Western Blot

Cells were collected in RIPA buffer (Sigma, R0278) for western blot analysis. Proteins were separated by SDS-PAGE (Mini-Protean TGX Precast Gels; Bio-Rad, Marnes-la-Coquette, France, 456-1083) and transferred to PVDF membranes (Bio-Rad, 170-4156). Blots were incubated with primary antibodies with 5% nonfat dry milk in TBS-0.1% Tween 20 overnight at 4 °C. HRP-linked secondary antibodies were used for 1 h at room temperature and antigen–antibody complexes were visualized by enhanced chemiluminescence (GE Healthcare, RPN2209; Luminol-based Enhanced Chemiluminescent (ECL) Western blotting detection reagents); quantifications were assessed using the ImageQuant LAS4000 imager software (GE Healthcare, Buc, France).

### 2.6. Antibodies

Anti-ACTB/β-ACTIN (clone 13E5, 4970S); GAPDH (clone D16H11, 5174S); MAP1LC3B (clone D11, 3868S); SQSTM1/p62 (5114S); ATG5 (clone D5F5U, 12994); ATG7 (2631); BECN1 (clone D40C5, 3495); and HRP-conjugated anti-rabbit and anti-mouse (7074S and 7076S) were purchased from Cell Signaling Technology. Mouse polyclonal anti-DUGV antibodies were obtained by hyper-immunization with DUGV (IRBA).

### 2.7. RNA Extraction and RT-qPCR Analysis

Viral RNAs were extracted either from infected-cell supernatant using QIAamp viral RNA mini kit (Qiagen, Courtaboeuf, France, 52906) or from infected cells using an RNeasy mini kit (Qiagen, 74106). Genomic strains were quantified using a quantitative one-step RT-PCR assay targeting the segment, as previously described [34], using iTaq Universal Probes Supermix (Bio-Rad, 172-5132) and Bio-Rad CFX96. Total RNAs were extracted from infected cells as mentioned above, and the expression of several mRNAs was assessed with the following forward and reverse primers (5′-3′): *MAP1LC3A/B* (TGTCCGACTTATTCGAGAGCAGCA, TGTGTCCGTTCACCAACAGGAAGA); *SQSTM1* (ATCGGAGGATCCGAGTGT, TGGCTGTGAGCTGCTCTT); *BECN1* (GAGGGATGGAAGGGTCTAAG, GCCTGGGCTGTGGTAAGT); *ATG3* (CCAACATGGCAATGGGCTAC, ACCGCCAGCATCAGTTTTGG); *ATG5* (TGAAAGGGAAGCAGAACCAT, TGCAGAAGGTCTTTTTCTGGA); *ATG7* (GCAAGCAAGAGAAAGCTGGT, AGGGTGGTTTGGACACAAGT); *ATG12* (CGAACCATCCAAGGACTCAT, CCATCACTGCCAAAACACTC); *GAPDH* (glyceraldehyde-3-phosphate dehydrogenase) (AGAAGGCTGGGGCTCATTT, CAGGAGGCATTGCTGATGA); *ACTB* (β-ACTIN) (CTCTTCCAGCCTTCCTTCCT, AGCACTGTGTTGGCGTACAG). Reverse transcription was carried out using the ImPromII Reverse Transcription kit (Promega, Charbonnières les Bains, France, A3800), iTaq universal SYBR Green Supermix (Bio-Rad, 170-8882) and Bio-Rad CFX96.

### 2.8. Fluorescence Microscopy

DUGV-infected and non-infected (Mock) GFP-LC3 HeLa cells were fixed for 20 min in 4% PFA after 48 h and permeabilized with PBS (Life Technologies, 10010-015)-0.5% Triton X-100 (Sigma, 3273372500) for 5 min. Cells were analyzed using confocal microscopy (LSM710 Zeiss) and the ImageJ software. The number of GFP+ dots was numerated from one single plan section per cell.

## 3. Results

### 3.1. Epithelial Cells Infected with DUGV Harbor an Augmented LC3 Lipidation Level and Accumulate LC3-Positive Punctiform Structures

**Figure 1:**
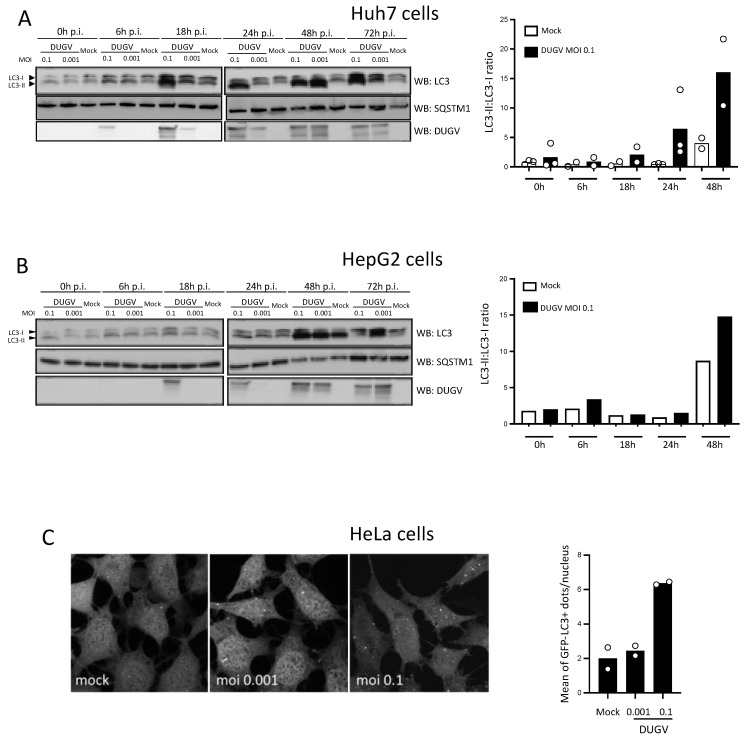
DUGV infection induces LC3 lipidation and biogenesis of LC3-positive punctiform structures in cultured epithelial cells. (**A**,**B**) Huh7 (**A**) and HepG2 (**B**) cells were infected with DUGV at MOI 0.1 and 0.001 or treated with virus-free supernatant (Mock) and analyzed by Western blot for the expression of LC3 (17 and 15 KDa), SQSTM1/p62 (SQSTM1), and DUGV proteins (DUGV) at different time points after infection. Quantification in A represents the LC3-II:LC3-I ratio for Huh7 cells. Values are the mean of two (18 h, 48 h) or three (0 h, 6 h, 24 h) independent experiments (circles indicate values of independent experiments). Quantification in B represents the LC3-II:LC3-I ratio for HepG2 cells from a single experiment representative of two. In some instances, the LC3-II:LC3-I ratio was not derived as the massive LC3-II signal made the calculation uncertain. (**C**) Confocal microscopy analysis of HeLa cells stably expressing a GFP-LC3 reporter construct 48 h after DUGV infection (MOI 0.1 and 0.001). GFP-LC3 puncta were quantified on plan sections. Results compiled from two independent experiments are expressed as the number of puncta per nucleated cell. At least 400 cells were examined for each condition (circles indicate values of independent experiments). Representative images from confocal microscopy are shown.

As the hepatocytic cell lines Huh7 and HepG2 were previously used to study the relationship between CCHFV infection and autophagy [32], we also used these cells to examine the level of conjugation of MAP1LC3/LC3 to phosphatidylethanolamine (conversion from LC3-I to LC3-II) during DUGV infection. A Western blot analysis that comprised six time points post-infection (0, 6, 18, 24, 48, and 72 h) and two multiplicities of infection (MOI) (0.1 and 0.001) revealed that, relative to non-infected cells, the level of LC3-II increased upon DUGV infection. In Huh7 cells infected at the MOI of 0.1, LC3-II was clearly augmented at 24 h post-infection and maintained up to 72 h post-infection (Figure 1A). Reducing the MOI to 0.001 delayed the induction of LC3-II in these cells. LC3-II induction was less marked in the case of HepG2 cells, another human hepatocytic cell line, with a substantial increase seen mostly at 48 h post-infection (Figure 1B). Because HeLa cells are a common tool for the study of mammalian autophagy, we also examined the status of LC3 in DUGV-infected HeLa cells. LC3-II accumulation was detectable and resembled that of HepG2 cells in terms of kinetics (data not shown). Of interest was the fact that the accumulation of LC3-II correlated well with the detectable expression of viral proteins in all analyses (Figure 1A,B). Additionally, we looked at the level of expression of SQSTM1, a long-lived protein that is degraded by autophagy and whose expression level may reflect autophagy flux activity, and found its level not to be substantially changed over time in Huh7- or HepG2-infected cells (Figure 1A,B). Because the viral infection of epithelial cells can, in some instances, trigger LC3 lipidation without initiating the biogenesis of autophagic vesicles [35], we examined whether the induction of LC3-II seen upon DUGV infection could correlate with an increase in the frequency of LC3-positive vacuolar structures. We infected HeLa cells stably expressing a green fluorescent protein (GFP)-LC3 reporter construct with DUGV and examined them via confocal microscopy for the presence of punctiform GFP-positive signals at 48 h post-infection. Of note, stable HeLa transfectants were used to minimize the presence of the artefactual GFP-LC3 signal which is often seen in experiments with transient transfectants due to LC3 incorporation into protein aggregates [36]. Unlike non-infected cells that displayed a limited number of LC3-positive puncta, i.e., the level of autophagosome associated with constitutive autophagy, cells infected with DUGV showed an accumulation of LC3-positive puncta that was clearly MOI-dependent (Figure 1C). The results thus show that DUGV infection causes the lipidation of the LC3 autophagy factor in epithelial cells and induces an accumulation of LC3-positive punctiform structures in GFP-LC3 reporter cells.

### 3.2. DUGV Infection Reprograms the Expression Level of Some Core Autophagy Genes

**Figure 2:**
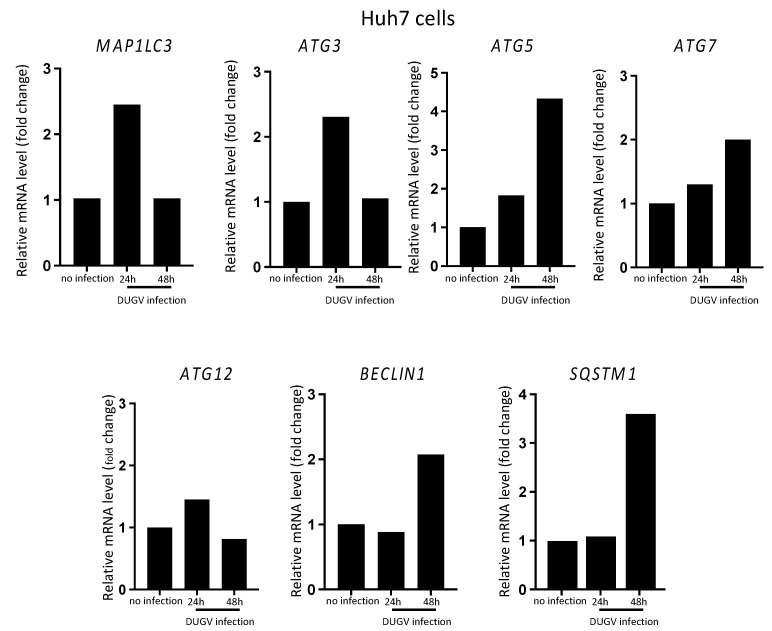
Profile of some autophagy-related gene expression during Huh7 cell infection with DUGV. Transcripts of *MAP1LC3A/B*, *ATG3*, *ATG5*, *ATG7*, *ATG12*, *BECLIN1*, and *SQSTM1* autophagy genes were quantified by RT-qPCR using total RNA from DUGV-infected Huh7 cells (MOI 0.01) at 0 (no infection), 24, and 48 h after infection. The house-keeping genes used were *GAPDH*, and *ACTB*. Fold changes in the mRNA level were calculated after deriving ΔΔCt and normalized to that of non-infected cells (mean of two independent experiments).

To examine whether the augmented lipidation of LC3 involved mainly the available pool of LC3-I proteins or whether it could relate to newly produced LC3 molecules generated through an augmented *MAP1LC3* gene transcription, we analyzed the level of *MAP1LC3A/B* transcripts by isolating total RNA and performing reverse transcription-coupled quantitative PCR (RT-qPCR) at 0, 24, and 48 h after DUGV infection. Concomitantly, we examined the expression profile of autophagy genes that are important for the process of LC3 lipidation (*ATG3*, *ATG5*, *ATG7*, *ATG12*, *BECN1*), as well as that of the gene encoding SQSTM1/p62 (*SQSTM1*), because cellular stress may in some instances modulate *SQSTM1* transcription. Relative to the expression levels observed at the time of infection (0 h post-infection), the transcripts of *MAP1LC3*, *ATG3*, and *ATG12* were upregulated at 24 h post-infection and returned to basal level by 48 h post-infection (Figure 2). *ATG5* and *ATG7* transcript levels were only slightly increased at 24 h post-infection, and this increase was essentially detected at 48 h post-infection. As for *SQSTM1* and *BECN1*, there was no change in transcription at 24 h post-infection, but there was a noticeable increase at 48 h post-infection. Thus, the transcription level of *MAP1LC3A/B* did fluctuate during DUGV infection, indicating that the pool of lipidated LC3 seen in virally infected Huh7 cells likely incorporates newly produced LC3 molecules. As for SQSTM1/p62, we observed a transient increase in the level of its transcript at 48 h post-infection. Along with the fairly stable level of the protein seen in the kinetic analysis (Figure 1A,B), the data suggest that SQSTM1/p62 degradation/recycling, and, therefore, the autophagy process itself, proceeds normally in DUGV-infected epithelial cells.

### 3.3. DUGV Infection-Associated LC3 Lipidation Involves ATG5/ATG7 Autophagy Factors

**Figure 3:**
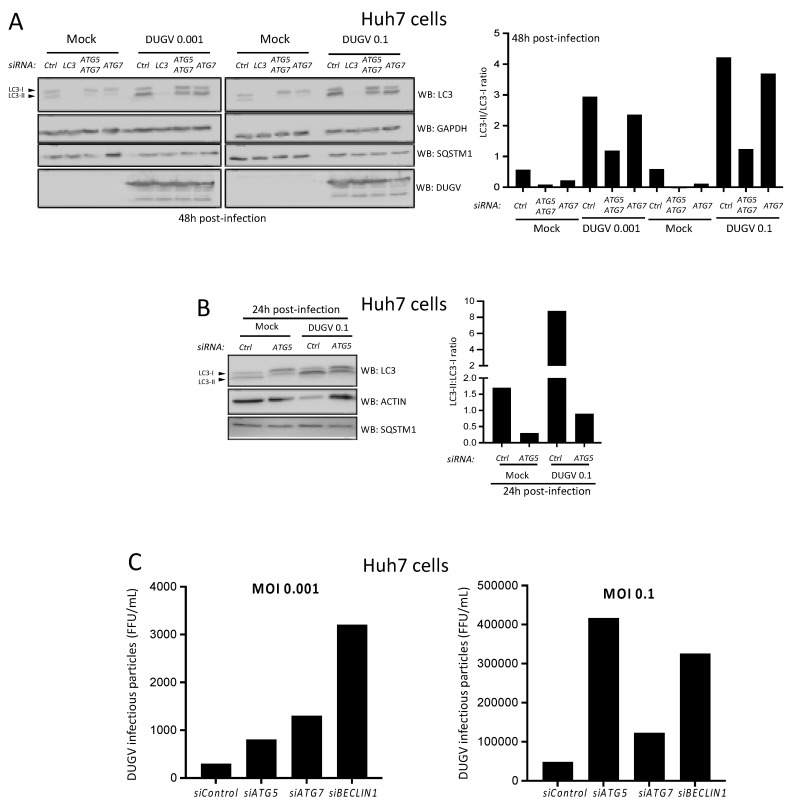
The influence of autophagy genes on DUGV infection-associated LC3 lipidation and virion production. (**A**,**B**) Huh7 cells were left uninfected (Mock) or infected with DUGV at MOI 0.1 and 0.001 for 48 h (**A**) or 0.1 for 24 h (**B**) in the presence of siRNA targeting either *ATG7*, *ATG5* plus *ATG7*, *MAP1LC3A-C* (LC3), or *ATG5* (along with control siCtrl) and analyzed by Western blot for the level of LC3 lipidation. Membranes were also examined for signals corresponding to GAPDH or ACTB, SQSTM1/p62 (SQSTM1), and DUGV proteins (DUGV). For each depicted blot, the associated quantification represents the LC3-II:LC3-I ratio. (**C**) Huh7 cells were treated with siRNA targeting *ATG5*, *ATG7*, or *BECLIN1* genes (versus siCtrl) for 48 h. Cells were then infected (MOI 0.1 and 0.001) with DUGV and the distinct conditions were analyzed for the amount of infectious viral particles produced after 48 h of infection. Values from titration in VeroE6 cells are expressed as focus-forming unit (FFU)/mL (one experiment for each MOI).

To analyze the role of the autophagy machinery in LC3-II formation during DUGV infection, the expression of the core autophagy gene *ATG7* was targeted for down regulation through the delivery of specific small interfering (si)RNAs. *ATG7*, or *ATG7* combined with *ATG5* for an optimal effect, were thus targeted for silencing for 48 h prior to infection at two MOI (0.1 and 0.001). Western blot analysis of non-infected cells indicated that both si*ATG7* and si*ATG7* + si*ATG5* affected the constitutive autophagy process, as the LC3-II:LC3-I ratio was reduced in both settings (Figure 3A). In the control experiment, we also silenced LC3 to verify that, in the absence of the LC3 factor, the whole LC3 signal would disappear in both control and DUGV-infected Huh7 cells. This was indeed the case, establishing that the LC3 profile seen during DUGV infection does not represent a cross-reactivity effect towards either a viral factor or a newly expressed cellular protein. In DUGV-infected cells, extinguishing *ATG7* alone mildly affected the level of LC3 lipidation. This inhibition effect on the LC3-II:LC3-I ratio was more pronounced when si*ATG7* and si*ATG5* were used in combination and could be seen at both the MOI used (Figure 3A). When the effect of si*ATG5* was examined individually, it confirmed that in DUGV-infected Huh7 cells, the augmented level of LC3 lipidation was to a significant extent dependent on ATG5 (Figure 3B). Thus, ATG5 and ATG7 core autophagy factors were involved in the augmented lipidation of LC3 observed during the DUGV infection of Huh7 cells.

### 3.4. DUGV Particle Production by Epithelial Cells Is Sensitive to Autophagy

Additional silencing experiments were conducted to delineate the role of autophagy on the outcome of Huh7 cell infection by DUGV. *ATG5*, *ATG7*, and *BECLIN1* were targeted for 48 h prior to infection (MOI 0.001 and 0.1), and the influence of the silencing on the capacity of Huh7 cells to produce infectious DUGV particles was assessed by running a plaque assay on VeroE6 cells with dilutions of culture supernatants that were collected 48 h post-infection. We noticed a trend towards a slight increase in the number of infectious DUGV particles produced by Huh7 cells infected at MOI 0.1 when *ATG5*, *ATG7*, or *BECLIN1* were silenced relative to control siRNA (Figure 3C). Consistent results were obtained at MOI 0.001. The results from gene silencing experiments thus point to a subtle negative influence of core autophagy genes on the production of infectious DUGV virions by Huh7 cells. This influence suggests that the autophagy machinery of Huh7 epithelial cells can exert a certain level of restriction on the production of viral particles.

## 4. Discussion

The orthonairovirus Dugbe is a tick-borne arbovirus able to cause clinical manifestations in humans. Very little information is available on how the virus replicates inside human cells in general and no knowledge is available on the relationship between DUGV and autophagic activity in particular. The present work represents the first attempt to gain insights into this issue, with a particular focus on epithelial cells. Under the conditions used, DUGV infection caused an MOI-dependent augmentation of the level of LC3 lipidation in hepatocytic epithelial cells, with a most noticeable effect clearly seen in Huh7 cells as soon as 18–24 h post-infection. Of note, enhanced LC3 lipidation was consistently associated with the detection of DUGV protein expression. DUGV infection also imposed an augmented biogenesis of LC3-positive punctiform structures in the cytosol of GFP-LC3-reporter cells in an MOI-dependent manner. These structures most likely correspond to the autophagosome, although an ultrastructural analysis would be necessary for their definitive characterization. These observations were well in line with those performed during the study of the infection of the same cells with CCHFV [32] and suggest that, for both viruses, the infection process has a mobilization effect on the autophagy machinery of epithelial cells.

The induction of LC3 lipidation was, however, more moderate in the case of DUGV as compared to CCHFV infection [32]. Yet, for both viruses, we observed that the level of LC3 lipidation was more pronounced in Huh7 cells than in HepG2 cells. Looking at cell line susceptibility to CCHFV infection, Dai et al. have observed that, based on the viral load detected in culture supernatants and viral nucleoprotein expression within cells, Huh7 cells were among the highly permissive cell lines, while HepG2 cells were classified as simply permissive [37]. HepG2 cells may produce less infectious particles than Huh7 cells, possibly due to an interferon (IFN)β competent status that is defective in Huh7 cells [38] and perhaps due to other differential gene expression [39]. We hypothesize that the higher induction of LC3-II seen in Huh7 cells relative to HepG2 cells, both for CCHFV and DUGV, may indeed relate to this differential permissiveness.

The induction of LC3-II is not rare during the viral infection of epithelial cells. The nature of this increased lipidation is not always well understood and it may or may not be required for the production of viral particles [35,40]. In the case of Huh7 cells infected with CCHFV, the massive level of LC3 lipidation was independent of ATG5 and ATG7 core autophagy factors and was also not reliant on the ATG3-ATG12 alternate pathway of lipidation, which can be recruited, for instance, during vaccinia virus infection [40], pointing to a novel non-canonical mode of lipidation. In contrast, the augmented LC3 lipidation seen during DUGV infection was sensitive to *ATG5*/*ATG7* gene silencing. This observation is compatible with the notion that the induction of LC3-II in DUGV-infected HuH7 cells involves the classical pathway of lipidation. However, one cannot completely exclude the possibility that some degree of non-conventional lipidation is also involved.

DUGV infection impacted the expression level of several core autophagy genes in Huh7 cells. Under the conditions used, we observed three types of modulation: (I) genes whose transcription level was upregulated only 48 h after infection (*SQSTM1*, *BECN1*); (II) genes whose expression was slightly upregulated at 24 h and further augmented at 48 h post-infection (*ATG5*, *ATG7*); and (III) genes harboring a transient up-regulation of transcription 24 h after infection (*ATG3*, *ATG12*, *MAP1LC3*). Although the analysis was performed at an intermediate MOI (0.01) and therefore may not completely reflect the situation corresponding to MOI 0.1, such a reprograming of autophagy gene expression is reminiscent of that observed during Huh7 infection with CCHFV with, however, some differences. Although the kinetic was comparable, CCHFV-induced increase in *MAP1LC3* expression was much higher than in the case of DUGV. The *SQSTM1* transcript was more rapidly and more intensively induced upon CCHFV infection and *BECN1* expression was more rapidly augmented as well [32]. Despite a certain degree of transcriptional activation at 48 h post-infection, the expression level of the long-lived autophagy protein SQSTM1 did not substantially vary over the course of DUGV infection and, in particular, did not progressively increase after infection. This was most consistent with the occurrence of a continuous flux of degradative autophagy, as SQSTM1 tends to massively accumulate when the autophagy flux is inhibited, for instance when viruses interfere with the autophagosome–endolysosome fusion process for their own benefit [41]. This is again consistent with what was observed in the case of epithelial cell infection by CCHFV [32].

Thus, despite material constraints that, in some instances, prevented us from running higher numbers of independent experiments, we believe the available results represents an important first step in delineating the relationship that evolved between DUGV infection and the autophagy process. We observed that in human epithelial cells, in vitro infection with the *Orthonairovirus* DUGV activates the autophagy machinery in an MOI-dependent manner and that such an activation tends to oppose viral replication. This was in contrast to CCHFV infection, for which we could not detect an impact of core autophagy gene engagement on virion production. Whether the marked endoplasmic reticulum (ER)-stress response [31] and hijacking of carbon and energy metabolism [42] that were uncovered in CCHFV-infected cells are also taking place in DUGV-infected cells and whether they impact the anti-viral autophagy of epithelial cells remain to be studied. In a comparative analysis of primary human antigen presenting cells (dendritic cells and macrophages derived from blood progenitors), DUGV was found to induce higher levels of cytokine and chemokine production (interleukin-6, RANTES, IP-10, and MIP-1α) than CCHFV, especially in the case of macrophages [30]. These differences in innate immune response by professional antigen presenting cells may contribute to the distinct pathogeneses associated with the two viruses. Along the same lines, we speculate that even with limited efficacy, the engagement of the autophagy machinery may participate in anti-DUGV cell autonomous defense and perhaps contributes to the moderate level of clinical manifestations during DUGV infection in humans.

## Data Availability

Not applicable.
